# GeSi Nanocrystals Photo-Sensors for Optical Detection of Slippery Road Conditions Combining Two Classification Algorithms

**DOI:** 10.3390/s20216395

**Published:** 2020-11-09

**Authors:** Catalin Palade, Ionel Stavarache, Toma Stoica, Magdalena Lidia Ciurea

**Affiliations:** 1 National Institute of Materials Physics, 405A Atomistilor Street, 077125 Magurele, Romania; catalin.palade@infim.ro (C.P.); toma.stoica@infim.ro (T.S.); 2 Academy of Romanian Scientists, 54 Splaiul Independentei, 050094 Bucharest, Romania

**Keywords:** optical sensor, photodetection of reflected light from asphalt, road conditions detection sensor, road safety, smart roads, k-nearest neighbor algorithm, artificial neural networks

## Abstract

One of the key elements in assessing traffic safety on the roads is the detection of asphalt conditions. In this paper, we propose an optical sensor based on GeSi nanocrystals embedded in SiO_2_ matrix that discriminates between different slippery road conditions (wet and icy asphalt and asphalt covered with dirty ice) in respect to dry asphalt. The sensor is fabricated by magnetron sputtering deposition followed by rapid thermal annealing. The photodetector has spectral sensitivity in the 360–1350 nm range and the signal-noise ratio is 10^2^–10^3^. The working principle of sensor setup for detection of road conditions is based on the photoresponse (photocurrent) of the sensor under illumination with the light reflected from the asphalt having different reflection coefficients for dry, wet, icy and dirty ice coatings. For this, the asphalt is illuminated sequentially with 980 and 1064 nm laser diodes. A database of these photocurrents is obtained for the different road conditions. We show that the use of both k-nearest neighbor and artificial neural networks classification algorithms enables a more accurate recognition of the class corresponding to a specific road state than in the case of using only one algorithm. This is achieved by comparing the new output sensor data with previously classified data for each algorithm and then by performing an intersection of the algorithms’ results.

## 1. Introduction

The car manufacturers, together with road authorities, are continuously challenged to supply effective services to ensure efficient and safe transportation. Fast and correct evaluation of slippery road conditions is critical for a driver, who should evaluate the conditions in order to have safe transportation and to prevent traffic accidents. The slow response of a human being is due to the limited information about road conditions that the human eye can provide and, therefore, using sensors for discriminating between dry, wet or icy asphalt is a requirement for the mitigation of traffic accidents.

At present, different types of technological systems, like fixed automated spray technology (FAST) [1,2], road weather information systems (RWIS) [3,4], environmental sensors [5], sensor networks for smart roads [6,7], pavement surface temperature sensors [8] and modern weather forecast systems [9], have been developed for detecting the asphalt state or for road monitoring under different atmospheric conditions. Great efforts have been made to integrate all these technological systems into a cooperative-intelligent transportation system (C-ITS) [10]. This platform was proposed to facilitate communications between vehicles and vehicles (V2V), vehicles and infrastructure (V2I) and infrastructure and vehicles (I2V) [11,12].

The development of sensor-based systems is the most appropriate solution with flexibility, portability and high applicability, which overcomes the limitations of the fixed systems as RWIS. Different sensor approaches were proposed in the study of Tabatabai et al. in which the authors proposed a sensor based on electrical resistance measurements that, besides determining roadway conditions (icy, dry, wet and frozen), transmits warnings about location and surface temperature [13]. Piccardi et al. studied different sensing geometries for the evaluation of the asphalt state using the measurement of the polarization/depolarization state of near infrared radiation (by two photodiodes). From their results, one can distinguish between a safe surface and different dangerous surfaces (wet, with water and icy) [14]. Jonsson et al. proposed another technique for determining road conditions based on infrared thermometry [15]. The measurements performed with a capacitive sensor represent another alternative proposed for detection of the road surface covered with water or ice. The limitation of this method is related to sensor contamination with dirt, fuel and salt [16,17,18]. In the paper published by Alimasi et al., the efforts are focused on a portable measurement system that can distinguish between different road states by comparing the ratio between specular reflectance and diffuse reflectance [19]. The recognition based on video images for identifying road conditions represents another approach proposed by Zhao et al. This method is based on road surface state recognition using a support vector machine (SVM) sustained by a grid searching algorithm and a particle swarm optimization algorithm (PSO) to improve recognition accuracy [20].

Using optical sensors to discriminate between different slippery road conditions is one of the best solution. Such an optical system is usually based on the comparison of the reflection coefficient at different wavelengths for different road states [14,15,21,22,23,24], for which broad-band optical sensors with extended sensitivity from visible (VIS) to short wave infrared (SWIR, 1–3 µm) are needed. Group IV, Si-Ge-Sn based photodetectors are very promising non-toxic alternatives to market available III-V devices. A cost-effective technique based on SiGeSn nanocrystals (NCs) embedded in an oxide matrix obtained by magnetron sputtering can be used for large-scale production of highly sensitive VIS-SWIR optical sensors [25,26,27,28,29,30]. In such composite materials, the oxide matrix has the role of the surface passivation of nanocrystals [28,29,31,32,33]. SiGe NCs alloys have the advantage of tuning the energy bandgap by adjusting the SiGe composition, in addition to the size controlling of the bandgap by quantum confinement effect. The SiGeSiO_2_ amorphous films deposited by magnetron sputtering and successively annealed by rapid thermal annealing (RTA) within the 700–1000 °C range for NC self-assembly, have shown photoresponsivity of about 5 AW^−1^ at room temperature, measured on coplanar diode structures [28].

In this paper we describe an optimized new optical sensor based on GeSi NCs embedded in a SiO_2_ matrix, that are fabricated by magnetron co-sputtering deposition of Ge, Si and SiO_2_ followed by thermal annealing, to be used for discriminating between different slippery road conditions in respect to dry asphalt. Such sensors based on eco-friendly materials, compatible with the well-established technology of Si, using cost-effective fabrication technology have many socio and economic advantages in respect to other sensors available on the market. The GeSi NCs sensors were experimentally tested by using them within an optical set-up for the detection of four types of road surface, namely dry, wet, icy asphalt and dirty ice asphalt. The experimental data were processed by two classification algorithms, namely k-nearest neighbor (KNN) and artificial neural network (ANN) algorithms for identifying the class to which road condition belonged. An important advantage of the proposed sensor is that this is also completely customizable for a better fit with the required needs. Thus, by changing the fabrication parameters, the spectral sensitivity of the sensor was adjusted in order to achieve a better matching between spectral characteristics of dry, wet and icy asphalt and the sensitivity domain of the sensor.

## 2. Materials and Methods

### 2.1. Optical Sensor Fabrication

The films with GeSiSiO_2_ were deposited by magnetron sputtering. The fabrication process was described elsewhere [27,28]. Here, we describe the layer fabrication procedure for optimized annealing. Thus, three separate targets (Ge, Si and SiO_2_) under DC and RF regime (20 W DC for Ge, 38 W DC for Si and 138 W RF for SiO_2_) were employed to ensure the Ge:Si:SiO_2_ composition of 24.2at%:27.4at%:48.4at% (corresponding to Si_x_Ge_1-x_ alloy in SiO_2_, with a concentration of x = 53at%). The deposition was made on n-type Si wafers chemically cleaned by a standard RCA(Radio Corporation of America) method in a Piranha solution and then covered by 50 nm SiO_2_ grown by dry oxidation in a rapid thermal processor (RTP) for electrical isolation of the GeSi NCs active layer from Si substrate. The as-deposited films have about 320 nm thickness.

The GeSi nanocrystallization was made by rapid thermal annealing (RTA) at optimal temperature of 900 °C for 10 min in N_2_ atmosphere. RTA temperature and time are critical in obtaining photosensitive GeSi NCs due to the competition between Ge fast diffusion and GeSi NCs formation in SiO_2_ matrix [25,26,27,28].

**Figure 1:**
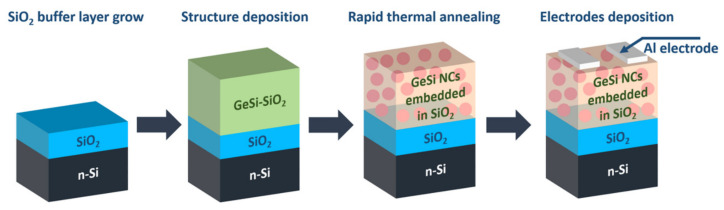
The workflow for obtaining the GeSi NCs:SiO_2_/SiO_2_/n-Si photodetector.

The workflow for obtaining the GeSi NCs:SiO_2_/SiO_2_/n-Si photodetector with Al contacts (magnetron sputtering) in planar geometry is presented in Figure 1.

### 2.2. Sensor Signal Measurements and Software Packet for Data Analysis

The spectral dependence of the sensor sensitivity was measured under monochromatic illumination with modulated light (120 Hz frequency) using an incandescent lamp (250 W) and Newport monochromator. For this, a lock-in amplifier (SR830) and a mechanical chopper (SR540) were used. For acquiring the photo-signal data for different states of the asphalt, the monochromatic light emitted by pulsed laser diodes of different wavelengths was measured after diffuse reflection on the asphalt surface using a homemade data acquisition system described below. For database analysis by discrimination algorithms, the Python 3.7 programming language was used. The software source codes and resulting data corresponding to the used KNN, ANN and comparing algorithms are given by Supplementary Materials.

## 3. Results and Discussions

### 3.1. Photodetector Characterization

**Figure 2:**
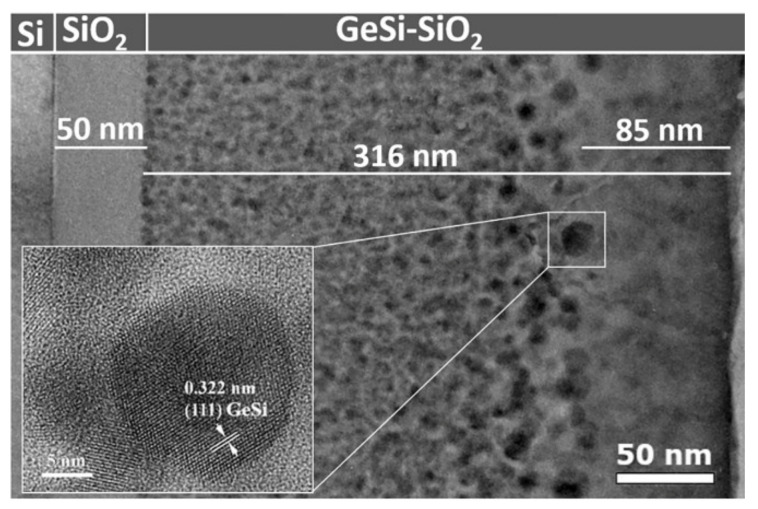
Low magnification XTEM image of GeSi NCs:SiO_2_/SiO_2_/n-Si structure. Inset is an HRTEM image of a spherical GeSi NC.

Figure 2 shows the cross-section TEM image at low magnification of GeSi NCs: SiO_2_/SiO_2_/n-Si structure. The image reveals the increase in density and the decrease in the size of the NCs towards the SiO_2_/Si substrate. This non-uniformity is caused by Ge and Si segregation and diffusion processes influenced by the depth-dependent internal stress in the film [34]. The upper part of the film of about 85 nm shows low Ge concentration due to the fast Ge diffusion and its surface oxidation and evaporation. By construction, the GeSi NCs:SiO_2_ layer is electrically isolated from the Si substrate by a 50 nm of SiO_2_ buffer layer.

The size of GeSi NCs increases from 5 nm at the bottom of the film to 18 nm at the surface. Figure 2 shows a low magnification XTEM (Cross-Sectional Transmission Electron Microscopy) image of the GeSi NCs:SiO_2_ film on SiO_2_/n-Si substrate. The inset in Figure 2 shows the HRTEM (High-Resolution Transmission Electron Microscopy) image of a spherical GeSi NC. The distance of 0.322 nm between (111) planes in GeSi NCs corresponds to about 40at% Si concentration as evaluated by linear interpolation, neglecting possible influence of strain in NCs. This value of Si concentration in GeSi NCs is lower than the mean concentration of about 53 at% Si of the SiGe alloy in the fabricated film.

**Figure 3:**
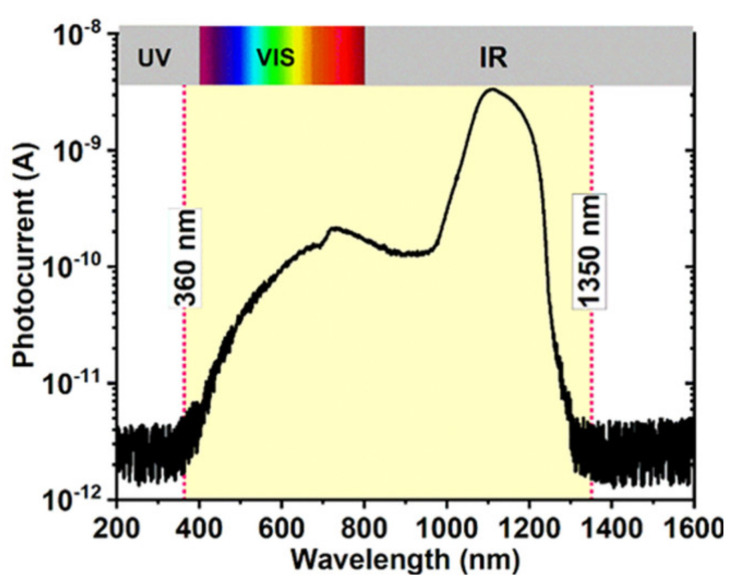
Spectral dependence of the photocurrent measured on GeSi NCs:SiO_2_ photodetector.

The non-uniform distribution of the NCs size is beneficial for broadening of the photosensitivity spectra. Figure 3 shows the photocurrent spectrum of the photodetector with spectral sensitivity detected in the range of 360–1350 nm.

The spectral distribution curve of the photocurrent presents a maximum at 1100 nm wavelength and another broad one in the 600–1000 nm range. The signal-noise ratio is 10^2^–10^3^.

### 3.2. Data Acquisition System

**Figure 4:**
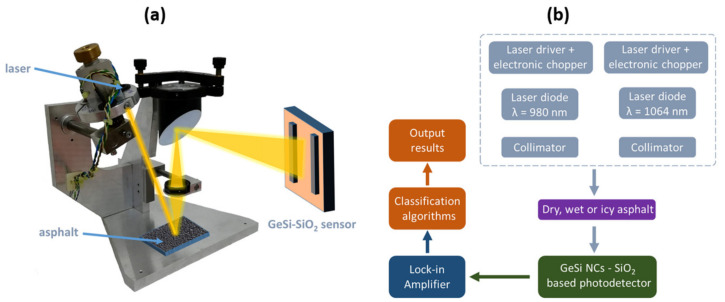
(**a**) The working principle and (**b**) the workflow of the sensor setup.

The working principle of the proposed optical sensor setup is based on different specular/diffuse reflections of the dry, wet and icy asphalt at different illumination wavelengths that is sketched in Figure 4a. Figure 4b shows the workflow of the sensor setup.

**Figure 5:**
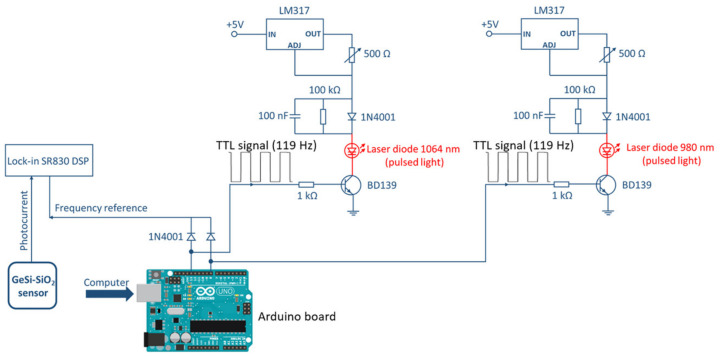
The electric circuit of the laser diode power supply.

Based on the spectral dependence of the photoresponse of GeSi NCs:SiO_2_ photodetector, we chose as the illumination source two laser diodes emitting about 200 mW in near-infrared (NIR), one laser diode RLT980-150GS of 150 mW from Roithner Lasertechnik with a wavelength of λ = 980 nm and another laser diode M9-A64-0200 of 200 mW from Thorlabs with a wavelength of λ = 1064 nm. The system is designed to be placed along the roads using an electric grid or independent renewable energy sources but could also be easily implemented in a novel integrated topology for electric vehicles [35]. The laser light and specular/diffuse reflections of light from the asphalt is collimated by using a collimator with a quartz lens and a parabolic mirror as shown in the Figure 4a. The power supply for the laser diodes was provided from a custom made constant current source circuit using a LM317 integrated circuit. The current was modulated through a BD139 transistor by using a TTL (Transistor–Transistor Logic) signal (0 ÷ 5 V) from an Arduino UNO board with a modulation frequency of 119 Hz. The electric circuit of the laser diode power supply is presented in Figure 5.

By modulating the current in the laser power supply, a pulsed laser light was obtained with the frequency of the TTL signal. The TTL signals measured by an oscilloscope are generated by an Arduino board and illustrated in Figure 5. Each laser diode has a separate power supply connected to the same Arduino board which gives the possibility to separately control the laser diodes from a computer connected to the Arduino board. The measurement procedure consists of the successive illumination of the asphalt at a precise angle with the two laser diodes such that only one laser diode is active at a time, then the reflected light is detected by the GeSi NCs:SiO_2_ photodetector. The photocurrent generated by the reflected light is then measured with a Stanford SR830 DSP lock-in amplifier that uses the frequency reference of the TTL signal generated by the Arduino board.

### 3.3. Optoelectronic Database for Different Asphalt States

**Figure 6:**
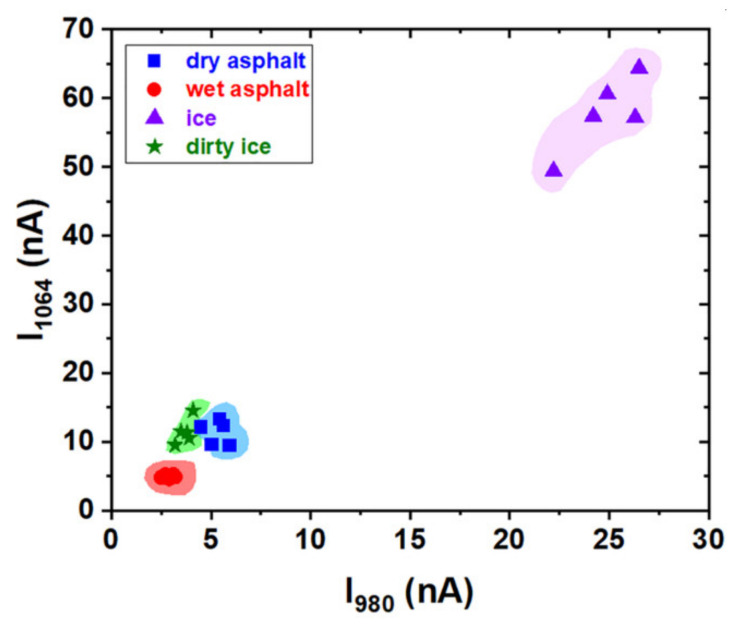
Experimental results obtained by multiple measurements of the photocurrent for the two laser diode illumination in the case of dry, wet, icy asphalt and dirty ice (frozen monolith of mixed asphalt powder, dust and water).

One complete measurement is done when the asphalt is successively illuminated with the two laser diodes. The photocurrent *I*_1064_, generated by the λ = 1064 nm wavelength illumination, is plotted as a function of the photocurrent *I*_980_, generated by the λ = 980 nm illumination. So, a complete determination represents one point in the *I*_1064_–*I*_980_ plot. The experimental results in Figure 6 were obtained by multiple measurements (in different zones of the asphalt) of the photocurrent for the two laser illumination for each case of dry, wet, icy asphalt and dirty ice (a mixture of asphalt powder, dust and water, frozen together). The asphalt was illuminated at an angle of 30 degrees related to normal incidence (Figure 4a). One can see that there is a good separation between the results corresponding to the different states of asphalt.

The best separation is obtained for icy asphalt for which one can observe that the photocurrent for both wavelengths (λ = 980 nm and λ = 1064 nm) is with order of magnitude higher than the photocurrent that corresponds to wet asphalt. In the case of dry asphalt, illumination with the λ = 980 nm wavelength generates a higher photocurrent compared with the one for dirty ice.

### 3.4. Database Analysis by Discrimination Algorithms

**Figure 7:**
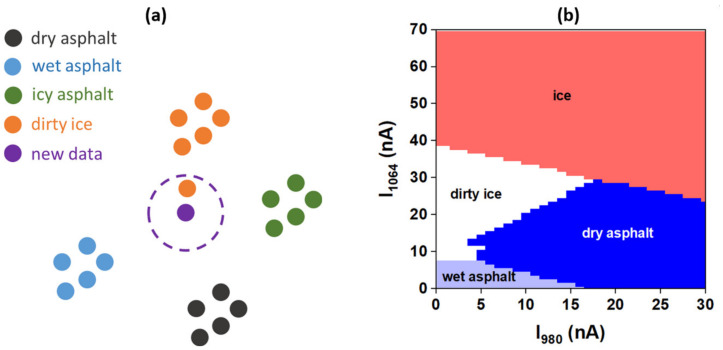
K-nearest neighbor (KNN) algorithm: (**a**) the working principle and (**b**) KNN algorithm applied to array data.

In order to have a system for complete detection of road conditions, it should be able to discriminate between dry, wet or icy asphalt. For this, first of all, separable clusters/classes of output data from the sensor ((*I*_1064_, *I*_980_) points), corresponding to each type of asphalt (dry, wet, icy asphalt and dirty ice), should be obtained. Additionally, it is necessary to have a classification algorithm to recognize the class by comparing the new output sensor data with previously classified data. For this, we used two different types of classification algorithms, namely k-nearest neighbor algorithm (KNN) and artificial neural network algorithm (ANN) [36]. For programming these algorithms, the Python 3.7 programming language was used and for the ANN algorithm, additionally, the Scikit-learn python [37,38] library was used. The KNN algorithm calculates the Euclidian distances in the *I*_1064_–*I*_980_ plane between the point that corresponds to new output sensor data and each point already classified from the measured photocurrents database and, finally, chooses the class to which the new point belongs considering the minimum distance (Figure 7a). We applied the KNN algorithm to our experimental data by generating an array of 30 × 70 points in the 0–30 nA and 0–70 nA ranges and classified them in Figure 7b.

**Figure 8:**
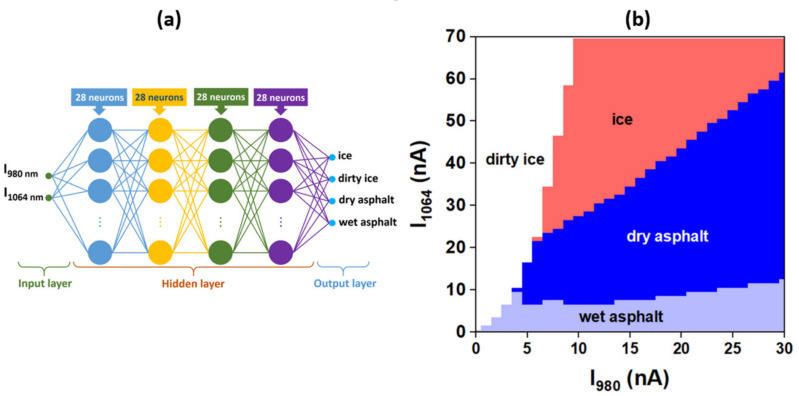
Artificial neural network (ANN) algorithm: (**a**) the working principle and (**b**) ANN algorithm applied to array data.

The advantage of the KNN algorithm over the ANN is that the KNN algorithm requires no training before using it in the classification procedure. However, the KNN drawback is that at higher database size the classification process becomes slower due to more iterations involved. Alternative fuzzy logic that is not investigated here could be based on the field programmable gate array (FPGA) or its Proportional-Integral-Derivative (PID)-type version [39]. The ANN algorithm works differently by simulating a network of artificial neurons that requires a training procedure [37] that is time consuming but at the end of the training procedure the algorithm works without training data making the classification very fast. We also applied the ANN algorithm to our experimental data, similarly to KNN algorithm. The proposed ANN configuration consists of two input neurons, four hidden layers (each one with 28 neurons) and four output neurons, the total number being 2520 synapses (weights). Each neuron in the network uses a rectifier linear unit (ReLU) as an activation function. The ANN algorithm was trained using the experimental data plotted in Figure 6 and after training, the network was fed with the same array as for the KNN algorithm. The results of applying the ANN algorithm on the array data and the working principle of the algorithm are presented in Figure 8.

**Figure 9:**
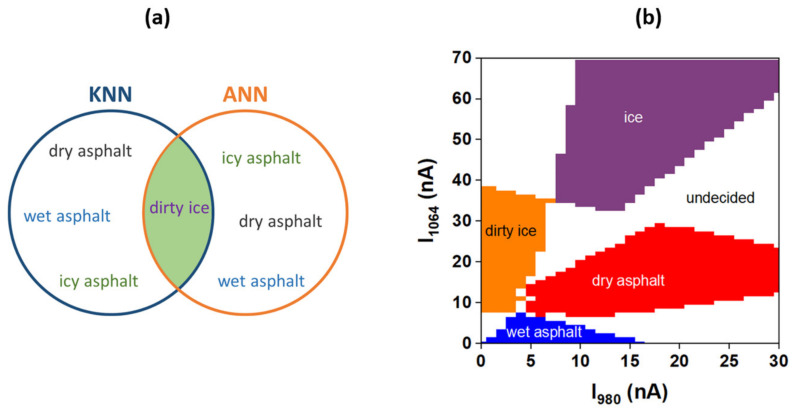
KNN and ANN algorithms’ classification intersection: (**a**) a schematic example of the intersection of the classification results of the KNN and ANN algorithms for the dirty ice state and (**b**) results of KNN and ANN intersection applied on the array data.

Both KNN and ANN algorithms provide a good mapping of the array data (data that arrive from the sensor). However, both algorithms have as a drawback the permanent correspondence between new data and the asphalt state. In other words, new data will always be assigned to one of the dry, wet, icy asphalt and dirty ice classes which is not always a correct decision due to the possibility of an asphalt mixed state occurring like wet-ice-dry asphalt or a new asphalt condition that needs to be learned. This drawback can be overcome by limiting the algorithms’ mapping around the experimental data and by introducing a new class that corresponds to undecided action which triggers the learning procedure of the ANN algorithm in order to assign the undecided point to an existing class or to create another one. This can be done by intersection of the classification results of the KNN and ANN algorithms. This means that the new data can be assigned to an existing class only if it is confirmed by both algorithms. If the asphalt class is confirmed by only one algorithm, then it will be assigned to an undecided class. The results of the algorithms’ intersection are presented in Figure 9. The software source codes and the resulting data corresponding to the KNN, ANN and comparing algorithms are given in the Supplementary Materials.

In reality, the undecided results correspond to an asphalt mixed state or to an unknown state which need to be covered by classification algorithms by labeling the data and training the algorithms. If a classification algorithm is trained to recognize a certain state of the asphalt, then the undecided results can be ignored because it is less probable for it to correspond to that specific state for which the algorithm is trained to recognize.

## 4. Conclusions

An optical sensor system based on GeSi NCs embedded in an SiO_2_ matrix was developed for discriminating between different slippery road conditions, namely wet, icy asphalt and dirty ice (frozen monolith of mixed asphalt powder, dust and water) in respect to dry asphalt. The sensor was fabricated by magnetron sputtering deposition of Ge, Si and SiO_2_ on oxidized n-type Si substrate, followed by RTA annealing for GeSi NCs formation. The photodetector has a spectral sensitivity in the range of 360–1350 nm and the signal-noise ratio is 10^2^–10^3^. The working principle of the sensor setup is based on the different reflection coefficients of the dry, wet and icy asphalt illuminated with 980 and 1064 nm laser diodes, one at a time. The experimental results show that the data obtained for different asphalt states present good separation and that it is possible to use classification algorithms, such as the k-nearest neighbor and artificial neural networks employed by us. Each classification algorithm provides excellent overlapping between experimental data and classified (predicted) ones but the mapping prediction falsely extends to infinity. This limitation is overcome by using the intersection of the classification results of the KNN and ANN algorithms leading to the constraining of the mapping prediction near the experimental data. The optical sensor together with the setup to be mounted along the road for road safety conditions is dedicated to be a potential platform for warning drivers with enough time and distance before reaching the slippery road. The proposed setup involves cheap materials, electronic components and fabrication processes. For future development of real cost-effective applications of the proposed road state detection platform, cheap light emitting diodes can be used. The present experiments have used lasers of less than 200 mW power, emitting in near infrared. Currently, the system is designed to be placed along the roads, ideally using renewable energy sources, but the proposed electro-optic detection system could be easily implemented in the future in a novel integrated topology for electric vehicles.

## Supplementary Materials

Software source codes and resulting data corresponding to the used KNN, ANN and comparing algorithms are available online at https://www.mdpi.com/1424-8220/20/21/6395/s1.
